# Breaking the Limitation of Elevated Coulomb Interaction in Crystalline Carbon Nitride for Visible and Near‐Infrared Light Photoactivity

**DOI:** 10.1002/advs.202201677

**Published:** 2022-06-02

**Authors:** Guoqiang Zhang, Yangsen Xu, Muhammad Rauf, Jinyu Zhu, Yongliang Li, Chuanxin He, Xiangzhong Ren, Peixin Zhang, Hongwei Mi

**Affiliations:** ^1^ College of Chemistry and Environmental Engineering Shenzhen University Shenzhen Guangdong 518060 P. R. China; ^2^ Institute of Information Technology Shenzhen Institute of Information Technology Shenzhen Guangdong 518172 P. R. China

**Keywords:** Coulomb interaction, crystalline carbon nitride, electron concentrations, near‐infrared response, n‐type doping

## Abstract

Most near‐infrared (NIR) light‐responsive photocatalysts inevitably suffer from low charge separation due to the elevated Coulomb interaction between electrons and holes. Here, an n‐type doping strategy of alkaline earth metal ions is proposed in crystalline K^+^ implanted polymeric carbon nitride (KCN) for visible and NIR photoactivity. The n‐type doping significantly increases the electron densities and activates the n→π* electron transitions, producing NIR light absorption. In addition, the more localized valence band (VB) and the regulation of carrier effective mass and band decomposed charge density, as well as the improved conductivity by 1–2 orders of magnitude facilitate the charge transfer and separation. The proposed n‐type doping strategy improves the carrier mobility and conductivity, activates the n→π* electron transitions for NIR light absorption, and breaks the limitation of poor charge separation caused by the elevated Coulomb interaction.

## Graphical abstract

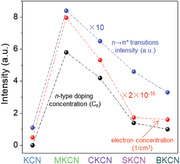

## 1 Introduction

Narrow bandgap photocatalysts exhibit extended visible and near‐infrared (NIR) light‐harvesting, thus improving the overall utilization efficiency of solar‐energy.^[^ ^1^ ^]^ During photocatalysis, the separation of photogenerated carriers is restricted by Coulomb interaction between electrons and holes.^[^ ^2^ ^]^ According to Coulomb's law: *F*_C_ = *kq*_1_ *q*_2_/*r* ^2^, where *F*_C_ is Coulomb force, *k* is Coulomb constant, *q*_1_ and *q*_2_ are the amount of charge, respectively, and *r* is the distance between valence‐band maximum (VBM) and conduction‐band minimum (CBM), so the *F*_C_ is inversely proportional to the square of the bandgap value.^[^ ^3^ ^]^ As a result, narrow bandgap photocatalysts, especially those with NIR response, are confronted with the bottleneck of strong Coulomb interaction.

Since the dissociated electrons and holes obey the Boltzmann distribution, the dissociation efficiency (DE) under Coulomb interaction can be calculated based on the formula “DE = e^–^ *^E^* ^C/(^ *^k^* ^B^ *^T^* ^)^”, where *E*_C_ is the binding energy of electrons and holes under Coulomb force, and *k*_B_ *T* is thermal excitation energy (≈25 meV) at room temperature.^[^ ^4^ ^]^ Accordingly, the DE of electrons and holes decreases sharply with the narrowing of bandgap, and photocatalysts with NIR response inevitably suffer from low charge separation.^[^ ^1^ ^]^ The top‐notch visible‐light responsive photocatalysts have an amazing apparent quantum efficiency (AQE) at 420 nm for photocatalytic H_2_ production, such as 93% of CdS^[^ ^5^ ^]^ and 50–70% of crystalline carbon nitride (CCN).^[^ ^6^ ^]^ However, most photocatalysts that can respond to 700 nm or NIR light, including the modified polymeric carbon nitride (CN),^[^ ^7^ ^]^ diethynylbenzene‐based polymers,^[^ ^8^ ^]^ black phosphorus complexes,^[^ ^9^ ^]^ sulfide heterojunctions,^[^ ^10^ ^]^ Au‐La_2_Ti_2_O_7_^[^ ^11^ ^]^ and elemental sulfur/phosphorus,^[^ ^12^ ^]^ exhibit an AQE of less than 25% at 420 nm. In brief, due to the inevitable elevated Coulomb interaction, most photocatalysts obtain NIR activity by sacrificing charge separation and visible light activity.

CCN has become a promising candidate for solar energy conversion because of its crystalline structure and superior performance to CN.^[^ ^13^ ^]^ Its intrinsic absorption band (less than 480 nm) originates in the *π*→π* electron transitions composed of the sp^2^ hybridization of C and N.^[^ ^6b^ ^]^ Other n→π* electron transitions larger than 500 nm are intrinsically weaker than *π*→π* transitions due to big differences in orbital electron densities.^[^ ^14^ ^]^ Considering that the conductivity depends on carrier concentrations and mobility, n‐type doping can greatly improve the carrier concentrations and conductivity of the samples,^[^ ^15^ ^]^ which is beneficial to the photogenerated carriers transport. In addition, the dramatically elevated electron densities may activate essentially prohibited n→π* electron transitions and even produce NIR light absorption.

Here, an n‐type doping strategy of alkaline earth metal ions is proposed in crystalline K^+^ implanted CN (KCN) for visible and NIR photoactivity. First, substituting divalent alkaline earth metal ions for monovalent K^+^ ions results in the conversion of additional valence electrons to free electrons, forming typical n‐type doping. Second, the alkaline earth metal elements are chemically closer to the alkali metal potassium, ensuring the low formation energy of this n‐type doping. Consequently, n‐type doping properties greatly improve the electron densities and activate the n→π* electron transitions, producing NIR light absorption. Although the elevated Coulomb interaction (10.8 meV vs 14.2 meV) reduces the DE of electrons and holes by 8.3%, the charge separation efficiency of n‐type doped samples is significantly higher than KCN due to the increase of carrier mobility by 2–4 times and conductivity by 1–2 orders of magnitude. This n‐type doping strategy can achieve NIR activity while still maintaining a relatively high visible light activity, breaking the limitation of the elevated Coulomb interaction.

## 2 Results and Discussion

### 2.1 Calculation and Analysis of Electronic Structures

**Figure 1:**
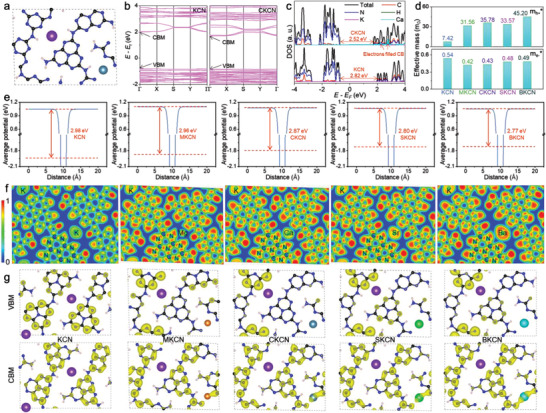
The optimized structures of a) alkaline earth metal ions doped KCN, b) electronic band structure, c) DOS, d) effective mass, e) work function, f) ELF, and g) VBM and CBM states. The atoms of C, N, K, and H are in black, blue, purple, and light pink, respectively. Alkaline earth metal atoms are orange (Mg), gray‐blue (Ca), green (Sr), and blue‐green (Ba).

The electronic structures of alkaline earth metal ions doped KCN are investigated through the plane‐wave technique implemented in the Vienna ab initio Simulation Package (VASP). The structural model of the crystallized K^+^ insertion into melon (C_12_N_18_H_6_K) proposed by Xu et al. is adopted.^[^ ^16^ ^]^ According to different ions (Mg^2+^, Ca^2+^, Sr^2+^, Ba^2+^) doping, the samples are named as MKCN, CKCN, SKCN, and BKCN, respectively. As shown in **Figure** **1**a, K^+^ and alkaline earth metal ions are inserted into 1D amine‐linked heptazine‐based melon chains. Taking Ca^2+^ ions doping as an example, the valence band (VB) of CKCN is more localized than KCN (Figure 1b). The calculated densities of states (DOS, Figure 1c) exhibit typical semiconductor properties, with the Fermi level at 0 eV located between the VBM and CBM. Previous reports have shown that alkaline earth metal ions can participate in forming inorganic semiconductor crystal structures, but have little effect on their energy band structures.^[^ ^17^ ^]^ Although alkaline earth metals are not directly involved in the formation of band edges, this *n*‐type doping increases overall electron densities. The electrons from alkaline earth metals partially fill the CB, lowering the CB position and thus narrowing the bandgap.

The effective masses of electrons and holes in units of electron rest mass (m_0_) are obtained by calculating the second derivatives at CBM and VBM.^[^ ^18^ ^]^ As shown in Figure 1d, after doping of alkaline earth metal ions, the electron effective mass decreases slightly, while the hole effective mass increases significantly, which is consistent with the more localized VB. In semiconductors, the effective mass of electrons is generally greater than that of holes, corresponding to the higher mobility of electrons than holes. Therefore, this regulation of greater difference in effective mass between electrons and holes is more favorable for the transmission and separation of photogenerated carriers.^[^ ^19^ ^]^

The *n*‐type doping is further elucidated by calculating work functions (Figure 1e). Alkaline earth metal ions doping makes more electrons transfer to the CB, thus bringing the Fermi energy level closer to the CB and reducing the corresponding work functions by 0.02–0.21 eV. The electron localization function (ELF) is employed to investigate the electron densities variation. The ELF is closer to 0, the more delocalized the electron. On the contrary, ELF is close to 1, indicating electron localization and a greater probability of occurrence position. As shown in Figure 1f, alkaline earth metals significantly elevated the electron densities in heptazine‐based melon chains, which is consistent with the work function calculations. Figure 1g exhibits the band‐decomposed charge densities of VBM and CBM. Compared with the symmetrical distribution in KCN, the distribution is more asymmetric and localized in *n*‐type doped samples. Due to the dramatically increased electron densities in melon chains around alkaline earth metals, VBM is localized in the region far away from alkaline earth metals. Such spatial separation of VBM and CBM states can facilitate the separation of photogenerated carriers.

### 2.2 Characterization of Morphology and Specific Surface Area

**Figure 2:**
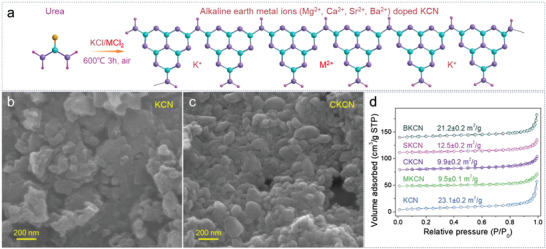
a) Synthetic procedure, b,c) SEM images, and d) the N_2_ adsorption–desorption isotherms.

The regulation of carriers effective mass as well as VBM and CBM states contribute to the transmission and separation of photogenerated carriers. In addition, the greatly increased electron densities may activate n→π* electron transitions, producing NIR light absorption.^[^ ^14^ ^]^ These impressing calculation results guide the exploration of alkaline earth metal ions doped KCN. Using KCl as a solid‐salt template can guide the ordered growth of CN in the confined space among KCl crystals during thermal polymerization, thus forming a highly crystalline KCN.^[^ ^7^, ^14^, ^16^ ^]^ In addition, K^+^ inserted between the amine‐linked heptazine‐based melon chains could be served as structural linkers to make melon chains more orderly arranged and form crystalline structures.^[^ ^20^ ^]^ Alkaline earth metal ions doped KCN samples were prepared in one‐step by adding corresponding chloride salts into KCl (**Figure** **2**a). Herein, urea will undergo a melting process (130–180 ℃) in thermal polymerization,^[^ ^21^ ^]^ which is conducive to homogeneous mixing with KCl and alkaline earth metal chlorides. CN is obtained under the same conditions by direct condensation of urea without KCl and chlorides. As shown in Figure S1, Supporting Information, the 10 g of urea can only produce 0.65 g of CN. After adding KCl, the yields are significantly increased to about 1.1 g. This may be due to the solid‐salt template effect of KCl, which makes the polymerization more complete.

The representative field emission scanning electron microscopy (FE‐SEM) images of the obtained samples are shown in Figure 2b,c, Figures S2a and S3, Supporting Information. CN presents an ultrathin lamellar structure, while KCN and doped KCN samples exhibit nanoparticles morphology of 100–500 nm. All the samples display ІІ‐type isothermal adsorption curves with H3‐type hysteresis loops (Figure 2d; Figure S2b, Supporting Information), indicating the mesoporous and macroporous structures. The BET specific surface areas are measured to be 62.0 ± 0.6, 23.1 ± 0.2, 9.5 ± 0.1, 9.9 ± 0.2, 12.5 ± 0.2, and 21.2 ± 0.2 m^2^ g^−1^ for CN, KCN, MKCN, CKCN, SKCN, and BKCN, respectively. The obvious changes in morphology and specific surface area between CN and KCN are caused by the crystallization structure induced by KCl. In addition, the specific surface area is related to the size and shape of particles, the agglomeration degree of particles and the stacking gap between particles, thus causing the difference of the specific surface area in KCN, MKCN, CKCN, SKCN, and BKCN samples. KCN and CKCN exhibit similar thermal stability, and both lose water before 200 ℃ and decompose violently after 600 ℃ (Figure S4, Supporting Information). The residual mass of the CKCN sample is larger than that of KCN at 800 ℃ due to Ca doping.

**Figure 3:**
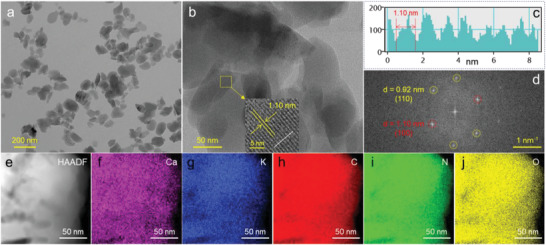
a,b) TEM images of CKCN; inset in (b) is the HR‐TEM image. c) The lattice fringes and d) FFT pattern in the inset of (b). e) STEM image and f–j) the corresponding elemental mappings.

The morphology of CN, KCN, and CKCN are further characterized by transmission electron microscopy (TEM). CN presents lamellar structure and no obvious lattice fringes are observed (Figure S5, Supporting Information). The KCN and CKCN samples exhibit nanoparticles morphology (**Figure** **3**a; Figure S6a, Supporting Information). In high resolution TEM (HR‐TEM) images (inset in Figure 3b; Figure S6c, Supporting Information), the clear lattice fringes are observed with a spacing of 1.10 nm, confirming the (100) crystal plane. Significant lattice fringes of 1.10 nm (Figure 3c) are also detected along the white line in the inset of Figure 3b. In addition, the diffraction points in the Fast Fourier Transform (FFT, Figure 3d) pattern further confirm the lattice spacings of 1.10 and 0.92 nm, corresponding to (100) and (110) crystal planes, respectively.^[^ ^16^ ^]^ Scanning TEM (STEM) with energy dispersive X‐ray is employed to investigate the elements distribution. The distribution of Ca, K, C, N, and O elements is relatively uniform in the whole region (Figure 3f–j), indicating that the Ca element is uniformly doped into the KCN structure.

### 2.3 Structural Characterization

**Figure 4:**
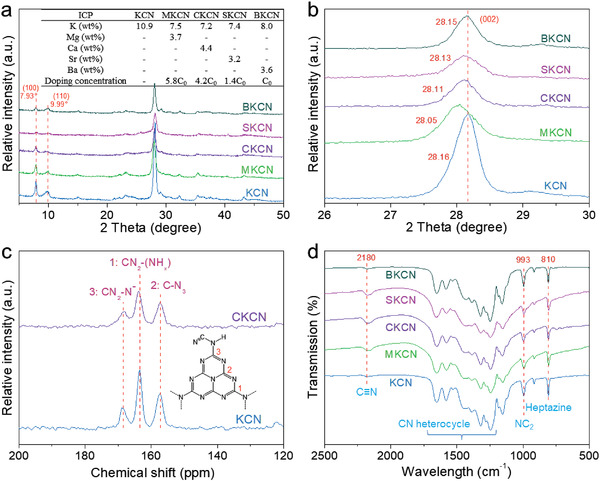
a,b) XRD patterns, alkaline earth metals content estimated by ICP‐OES (inset in (a)), c) solid‐state CP/MAS ^13^C NMR spectra and d) FTIR spectra.

The existence of corresponding alkaline earth metal elements, with the contents between 3.2–4.4 wt%, is confirmed by inductively coupled plasma optical emission spectrometry (ICP‐OES, inset in **Figure** **4**a). The decrease of K contents in n‐type doped samples can be attributed to the substitution of K by alkaline earth metals. The doping concentration can be calculated according to the mass fraction and relative atomic mass of elements. The Mg, Ca, and Sr concentrations in MKCN, CKCN, and SKCN are 5.8C0, 4.2C0, and 1.4C0, respectively, when the doping concentration of Ba in BKCN is taken as C0. The X‐ray diffraction (XRD) patterns are shown in Figure 4a,b and Figure S7, Supporting Information. In contrast to CN, KCN presents three significantly narrowed diffraction peaks at 7.93°, 9.99°, and 28.16° due to the crystalline structure, corresponding to (100), (110), and (002) crystal planes, respectively.^[^ ^16^ ^]^ The intensity of these three peaks decreases with alkaline earth metal ions doping while retaining the typical KCN structure. Compared to KCN, MKCN has shown the biggest shift (0.11°) in the main peak due to the high doping concentration. On the contrary, BKCN exhibits the smallest peak shift (0.01°). The KCN doped with different contents of Ca^2+^ ions are further investigated through XRD analysis. As shown in Figure S8, Supporting Information, the main peak gradually weakens and shifts to a lower angle with the increase of Ca^2+^ ions doping.

Elemental analysis shows that the C/N molar ratios are 0.696 and 0.699 in KCN and CKCN samples, with no significant change (Table S1, Supporting Information). The solid‐state CP/MAS ^13^C nuclear magnetic resonance (NMR) are conducted to analyze possible structures and skeletons, as shown in Figure 4c. Using KCN and CKCN as examples, the three NMR peaks at 157.0, 163.4, and 168.4 ppm show the presence of carbon atoms in C—N_3_, CN_2_—(NH*_x_*), and CN_2_—(N^–^) units,^[^ ^22^ ^]^ confirming the heptazine‐based skeletons. Fourier transform infrared spectroscopy (FTIR, Figure 4d) is adopted to characterize the characteristic structures. The fingerprint peaks at 1200–1700 cm^−1^ correspond to the stretching and bending vibrations of aromatic heterocycle,^[^ ^23^ ^]^ while the signal at 2180 cm^−1^ belongs to the stretching vibration of the cyano group (C≡N). The peak at 810 cm^−1^ is ascribed to the out‐of‐plane bending vibration of heptazine rings. Besides, the signal at 993 cm^−1^ could be assigned to the symmetric and asymmetric vibrations of NC_2_ bonds in metal‐NC_2_ units,^[^ ^6^, ^24^ ^]^ indicating that K^+^ and alkaline earth metal ions are implanted between the heptazine‐based melon chains.

**Figure 5:**
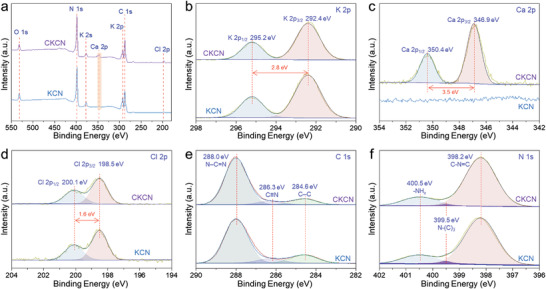
a) The survey XPS spectra and b–f) high‐resolution XPS spectra of KCN and CKCN.

X‐ray photoelectron spectroscopy (XPS) measurements are performed to characterize chemical structures. In both KCN and CKCN samples, the survey XPS spectra (**Figure** **5**a) reveal the existence of C, N, O, and K elements, but only the Ca element exists in CKCN. In Figure 5b, the K 2p_1/2_ and K 2p_3/2_ peaks with a spin‐orbit splitting of 2.8 eV are observed at 295.2 and 292.4 eV, respectively, belonging to the K^+^ ion.^[^ ^25^ ^]^ Figure 5c exhibits the high‐resolution Ca 2p spectrum, the peaks at 350.4 (2p_1/2_) and 346.9 eV (2p_3/2_) with a spin‐orbit splitting of 3.5 eV are assigned to the Ca^2+^ ion.^[^ ^26^ ^]^ The presence of Cl^–^ ion (≈0.5 at%) observed by XPS analysis, possibly from surface adsorption, is evidenced by the Cl 2p_1/2_ and Cl 2p_3/2_ peaks with a spin‐orbit splitting of 1.6 eV at 200.1 and 198.5 eV (Figure 5d), respectively.^[^ ^27^ ^]^ The C 1s XPS can be fitted into three peaks at 288.0, 286.3, and 284.6 eV (Figures 5e; Figure S9b, Supporting Information), belonging to C atoms in aromatic N—C═N units,^[^ ^28^ ^]^ C≡N and graphitic carbon,^[^ ^29^ ^]^ respectively. Besides, the N 1s XPS exhibits three contributions, located at respectively 398.2, 399.5, and 400.5 eV (Figure 5f; Figure S9c, Supporting Information), which could be attributed to N atoms in C—N═C, N—(C)_3_ and bridging —NH*_x_* or C≡N species, respectively.^[^ ^28^ ^]^ According to the proportion of integral area, the C—N═C component in N 1s XPS is 86.5% and 86.3% of KCN and CKCN samples respectively, indicating no obvious change of heptazine‐based skeletons. In addition, their C—N═C components is significantly higher than 76.7% in CN, probably due to the solid‐salt template effect of KCl, which makes the heptazine polymerization more complete. The above characteristics confirm the successful doping of alkaline earth metal ions while maintaining the typical KCN crystalline structure.

### 2.4 Characterization of Carriers and Absorption Characteristics

The n‐type doping of alkaline earth metal ions reduces and improves the effective mass of electrons and holes respectively, and makes the spatial separation of VBM and CBM states, which is beneficial to the transmission and separation of photogenerated carriers. The Hall effect is an electromagnetic effect that provides information such as resistivity, conductivity type, carrier mobility and carrier concentration of samples. The van der Pauw method can be applied to the sheet samples with uniform thickness and arbitrary shape. During testing, the shape factor (*f*) should be controlled at about 1. As shown in **Table** **1**, the negative Hall coefficients indicate that these samples are all typical n‐type semiconductors and the carriers are mainly electrons,^[^ ^30^ ^]^ consistent with the calculation of work functions (Figure 1e) and ELF (Figure 1f). For n‐type doped semiconductors, the conductivity and electron concentration highly depend on the doping concentrations. The resistivity of the samples is between that of semiconductors (10^–1^–10^9^ Ω cm), and the conductivity increases by 1–2 orders of magnitude after n‐type doping. In MKCN and CKCN samples, the electron concentration increases considerably by an order of magnitude. More importantly, the carrier mobility of the doped samples exhibits a two‐ to fourfold increase than KCN. These results further confirm the improvement of electron concentration and mobility, as well as conductivity by n‐type doping of alkaline earth metal ions.

**Table 1:** The measured resistivity, Hall coefficient, carrier concentration, and carrier mobility using a van der Pauw Hall measurement system

	Resistivity [Ω cm]	Hall coefficient [cm^3^ C^−1^]	Carrier concentration [1 cm^−3^]	Carrier mobility [cm^2^ (V s)^−1^]	*f*‐factor
KCN	18 600	‐4386	2.45 × 10^15^	0.136	0.9647
MKCN	278	‐1824	3.98 × 10^16^	0.548	0.9718
CKCN	686	‐1772	2.66 × 10^16^	0.588	0.9516
SKCN	1765	‐2410	8.67 × 10^15^	0.406	0.9487
BKCN	2819	‐3049	8.03 × 10^15^	0.333	0.9617

**Figure 6:**
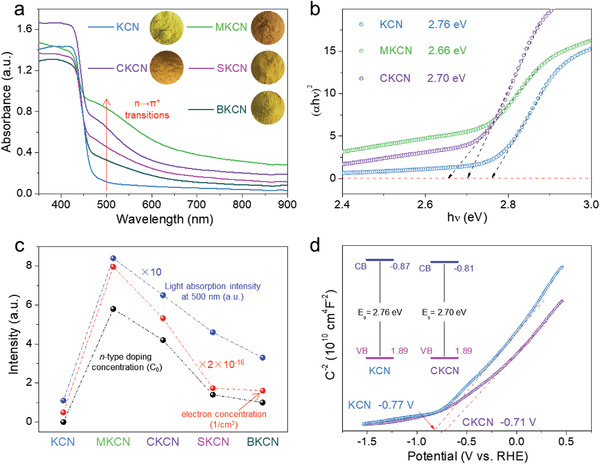
a) The UV–vis–NIR DRS spectra and b) Tauc plots. Insets in (a) are the optical photos. The relationship of n‐type doping concentration, electron concentration, and light absorption intensity at 500 nm for c) KCN, MKCN, CKCN, SKCN, and BKCN samples. d) Mott–Schottky plots and band position diagram (inset).

In addition, the n‐type doping of alkaline earth metal ions increases the electron densities and makes the electrons partially fill the CB, thus lowering the CB position and narrowing the bandgap. The light absorption expands due to the activated n→π* electron transitions,^[^ ^14^ ^]^ resulting in obvious NIR absorption (700–900 nm) in the ultraviolet‐visible‐near infrared diffuse reflectance spectra (UV–vis–NIR DRS, **Figure** **6**a). Among them, MKCN shows the largest light absorption expansion, followed by CKCN, while the absorption of SKCN and BKCN improves relatively small. The color of the samples significantly deepens and turns brown in MKCN (inset in Figure 6a). As shown in Figure S11, Supporting Information, the color of the CKCN series samples gradually deepens to orange‐red as the Ca^2+^ ion doping increases. We tried to doping CN with alkaline earth metals in the absence of potassium, without obvious light absorption expansion, probably due to the crucial role of potassium in n‐type doping. Tauc plots (Figure 6b) demonstrate that the bandgap narrows by around 0.10 and 0.06 eV following Mg^2+^ and Ca^2+^ ions doping, respectively, compared to 2.76 eV for KCN.

Considering that the peak value of n→π* electron transitions in Figure 6a is mainly around 500 nm, we investigated the relationship between the light absorption intensity of 500 nm, and the electron and doping concentrations. After n‐type doping, the Fermi level (*E*_F_′) can be calculated by formula “*E*_F_′ = *E*_F_ + *k*_B_ *T*(ln*N*_D_/*N*_e_)”, where *E*_F_ and *N*_e_ are the Fermi level and electron concentration before doping, respectively, *k*_B_ is Boltzmann constant, *T* is temperature, and *N*_D_ is doping concentrations.^[^ ^31^ ^]^ Thus, the electron concentration depends on the concentration of n‐type doping. As shown in Figure 6c, the variation of electron concentration and n→π* electron transitions in KCN, MKCN, CKCN, SKCN, and BKCN samples is consistent with the doping concentrations. Therefore, the electron concentration can be greatly increased to activate and produce stronger n→π* electron transitions by regulating the n‐type doping concentrations.

Mott–Schottky analysis is used to determine the semiconductor type and flat band position of KCN and CKCN samples. As illustrated in Figure 6d, the slope of the linear part in two curves is positive, indicating the typical n‐type semiconductor characteristics. The flat band potentials (*E*_fb_) of KCN and CKCN are determined as −0.77 and −0.71 V versus reversible hydrogen electrode (RHE) by the intercepts with the potential axis. Considering that the CB potential of an *n*‐type semiconductor is generally considered to be 0.1–0.2 V higher than *E*_fb_, the potential difference here is set at 0.1 V. The CB positions of KCN and CKCN are −0.87 and −0.81 V versus RHE, respectively, while the VB positions are obtained by adding CB to the bandgap value. The band position diagram (inset in Figure 6d) indicates that the CB position is lowered after Ca^2+^ ions doping, consistent with the band structure (Figure 1b) and DOS (Figure 1c) calculations. The VB position has no obvious change, matching the VB‐XPS analysis (Figure S12, Supporting Information).

### 2.5 Analysis of Carrier Coulomb Interaction, Transport, and Separation

**Figure 7:**
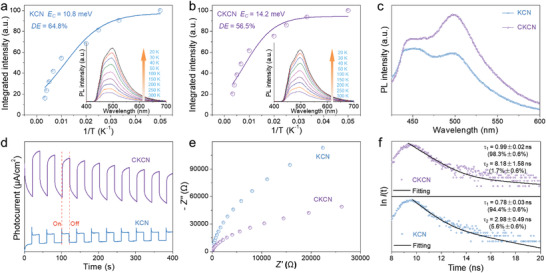
TD‐PL spectra (insets in (a, b)) from 20 to 300 K under 325 nm laser excitation. a,b) Integrated PL emission intensity as a function of temperature from 20 to 300 K. c) The steady‐state PL, d) visible‐light photocurrent measurement, e) EIS and f) TRPL.

The Coulomb interaction can be well investigated by measuring the binding energy of electrons and holes under Coulomb force via temperature‐dependent photoluminescence (TD‐PL). The *E*_C_ value is fitted by the formula “*I*(*T*) = *I*_0_/(1 + *Ae* ^–^ *^E^* ^C/(^ *^k^* ^B^ *^T^* ^)^)”, where *I*_0_ is PL strength at 0 K and *k*_B_ is Boltzmann constant.^[^ ^32^ ^]^ As shown in insets of **Figure** **7**a,b, the increase of temperature provides a driving force for electrons and holes dissociation, thus reducing PL strength. The *E*_C_ of CKCN is calculated to be 14.2 meV, which is 3.4 meV higher than KCN (Figure 7a,b), demonstrating the elevated Coulomb interaction in CKCN. According to “DE = e^–^ *^E^* ^C/(^ *^k^* ^B^ *^T^* ^)^” (*k*_B_ *T* ≈ 25 meV at room temperature),^[^ ^4^ ^]^ the DE of electrons and holes in KCN and CKCN are estimated about 64.8% and 56.5%, respectively. Since the elevated Coulomb interaction leads to more undissociated electrons and holes (43.5% vs 35.2%), CKCN presents a stronger fluorescence emission than KCN in steady‐state PL (Figure 7c).

Although the DE in CKCN reduces by 8.3%, it exhibits significantly increased visible‐light photocurrent signal (Figure 7d) and charge separation efficiency than KCN due to quadrupled carrier mobility and two orders of magnitude higher conductivity. The CKCN presents a smaller semicircle radius of the Nyquist plots according to electrochemical impedance spectroscopy (EIS, Figure 7e) measurements, further indicating better charge transfer than KCN. The PL decay in time‐resolved photoluminescence (TRPL, Figure 7f) is well fitted by the double exponential function, which can be assigned to these two processes as the faster decay in bulk and the slower decay in surface trap states, respectively.^[^ ^33^ ^]^ The lifetime of *τ*_1_ and *τ*_2_ is significantly higher in CKCN than in KCN, probably due to the elevated Coulomb interaction, making the PL decay slower. In addition, the relative weight of the surface recombination process is reduced from 5.6% ± 0.6% for KCN to 1.7% ± 0.6% for CKCN, which may be due to the reduced specific surface areas.^[^ ^33^ ^]^

**Figure 8:**
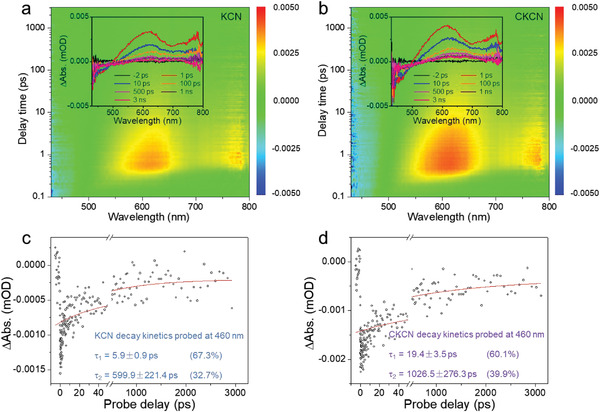
a,b) Representative TA spectra at different probe delays (insets) and time‐wavelength‐dependent TA color maps (pump at 330 nm). c,d) TA decay kinetics probed at 460 nm.

The ultrafast femtoseconds transient absorption (fs‐TA) spectroscopy is performed to illustrate the dynamics of photogenerated carriers.^[^ ^34^ ^]^ Two samples are excited from the ground state to the excited state at 330 nm pump light. Continuous ground state bleaching and excited state absorption signals are detected between 430–800 nm (**Figure** **8**a,b and insets). The CKCN sample presents a stronger TA signal than KCN, indicating more active charges generation and separation. The decay dynamics of photogenerated carriers probed at 460 nm can be well fitted by a double‐exponential function, which corresponds to the traps of CB electron relaxation to different depths (Figure 8c,d).^[^ ^35^ ^]^ In CKCN, the shorter lifetime *τ*_1_ (19.4 ± 3.5 ps) and the longer lifetime *τ*_2_ (1026.5 ± 276.3 ps) are significantly extended than those of KCN (*τ*_1_ = 5.9 ± 0.9 ps; *τ*_2_ = 599.9 ± 221.4 ps), consistent with the TRPL results. This extended excited‐state carrier lifetime gives carriers a greater chance to participate in photocatalytic reactions. These results demonstrate that this n‐type doping breaks the limitation of poor charge separation caused by the elevated Coulomb interaction.

### 2.6 Characterization of Photocatalytic Activity

The regulation of light absorption and carrier mobility by n‐type doping of alkaline earth metal ions is expected to exhibit excellent photocatalytic performance. Using triethanolamine (TEOA) as a sacrificial agent and 3 wt% Pt as a co‐catalyst, the photocatalytic H_2_ production activity is evaluated under visible and NIR light irradiations. The reaction temperature is controlled to 5 ℃ by the recirculating cooling water system. Since the concentration of photocatalysts affects the light penetration depths, the photocatalytic performance improves with the increase of photocatalyst amounts, and finally remains unchanged. The activity remains unaffected when the photocatalyst amounts are more than 50 mg, as shown in Figure S13, Supporting Information, so the mass of the optimized photocatalysts is fixed at 50 mg.

**Figure 9:**
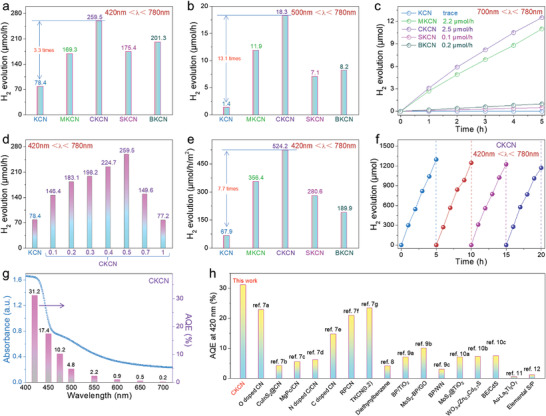
H_2_ production of KCN and alkaline earth metal ions doped KCN under a) 420 nm < *λ* < 780 nm, b) 500 nm < *λ* < 780 nm, and c) 700 nm < *λ* < 780 nm irradiations. d) H_2_ production rates of CKCN with different contents of Ca^2+^ ions doping. e) Normalized H_2_ production rates of specific surface areas. f) The recycling measurements of the H_2_ production. g) Wavelength‐dependent AQE under monochromatic light irradiations. h) Comparison of AQE at 420 nm between CKCN and the reported first‐rate photocatalysts capable of responding to 700 nm or NIR light.

As shown in **Figure** **9**a and Figure S14, Supporting Information, the H_2_ production rate for 50 mg of CN and KCN samples were 23.9 and 78.4 µmol h^−1^ (420 nm < *λ* < 780 nm). Due to the crystallization structure, the activity of KCN is significantly higher than that of CN. With the doping of alkaline earth metal ions, the activity increased significantly. Among the different samples, CKCN and BKCN presented the high activity of 259.5 and 201.3 µmol h^−1^, which were 3.3 and 2.6 times that of KCN, while MKCN and SKCN samples showed relatively high activity of 169.3 and 175.4 µmol h^−1^. Extended light absorption allows exploration of longer wavelength photocatalytic performance. Figure 9b shows that CKCN has a high activity of 18.3 µmol h^−1^ (500 nm < *λ* < 780 nm), which is 13.1 times higher than KCN (1.4 µmol h^−1^). Other samples, such as MKCN, SKCN, and BKCN, had an activity that was 8.5, 5.1, and 5.9 times higher than that of KCN. More importantly, NIR (700 nm < *λ* < 780 nm) photoactivity is generated after doping, for example, CKCN has an activity of 2.5 µmol h^−1^, while KCN is not active (Figure 9c).

CKCN presents the highest carrier mobility among all alkaline earth metal ions n‐type doped samples, which is beneficial to the photogenerated carriers transport. In addition, compared with KCN, its light absorption is significantly expanded at both greater than 500 and 700 nm, slightly lower than the highest MKCN. As a result, CNCN exhibits the highest H_2_ evolution activity in all doped samples at three different test bands (420–780 nm, 500–780 nm, and 700–780 nm). The optimization of Ca^2+^ ions doping amounts is shown in Figure 9d. The increase in doping amounts significantly improved H_2_ production activity, but excessive doping will result in a decline. Specific surface area is one of the important factors affecting photocatalytic activity. In order to exclude the effect of specific surface areas, the H_2_ production rates normalized by specific surface areas were investigated. As shown in Figure 9e, the normalized activity after doping also presents a significant upward trend compared with KCN. The CKCN sample exhibits the highest activity of 524.2 µmol h^−1^ m^−2^, 7.7 times that of KCN (67.9 µmol h^−1^ m^−2^). After four cycles of photocatalytic reaction, that is, 20 h, the activity only decays by 9.7% in CKCN (Figure 9f). In addition, there was no obvious change in XRD patterns before and after photocatalytic reactions (Figure S15, Supporting Information), which further explained the relatively excellent photostability of CKCN.

The AQE is measured using different monochromatic light irradiations (Figure 9g). The AQE of CKCN at 420 and 500 nm is 31.2% and 4.8%, respectively. Even at 700 nm, its AQE is still 0.2%. As the bandgap narrowing is inevitably accompanied by the sharply elevated Coulomb interaction between electrons and holes, photocatalysts that can respond to 700 nm or NIR light suffer from low charge separation. As shown in Figure 9h, their AQE at 420 nm are less than 25%, far below that of visible‐light responsive photocatalysts, such as 93% of CdS^[^ ^5^ ^]^ and 50–70% of CCN.^[^ ^6^ ^]^ Although the AQE of CKCN decays sharply beyond 450 nm, its AQE at 420 nm is one of the highest levels among all photocatalysts, with a wide spectral response of more than 700 nm. In addition, similar to AQE calculations, we use xenon lamp equipped with AM 1.5G filter to simulate sunlight to measure the solar to hydrogen conversion efficiency (STH). The average optical power density is adjusted to 100 mW cm^−^^2^ and the equivalent wavelength of sunlight spectrum is chosen as 584.3 nm based on GB/T 26915‐2011. The STH is calculated as 0.02%, 0.07%, and 0.28% for CN, KCN, and CKCN samples, respectively. Such excellent activity can be attributed to the regulation of light absorption, carrier mobility and conductivity by n‐type doping, breaking the limitation of the elevated Coulomb interaction.

## 3 Conclusions

Alkaline earth metal ions n‐type doped KCN were prepared for visible and NIR photocatalytic H_2_ production. The n‐type doping activates the n→π* electron transitions by increasing the overall electron densities, thus producing NIR light absorption. Their electron concentrations and light absorption intensity can be regulated by n‐type doping concentrations. In addition, the regulation of carriers effective mass as well as VBM and CBM states is beneficial to charge transfer and separation. Consequently, the photoactivity under visible and NIR light is greatly enhanced after doping. With the improvement of carrier mobility and conductivity, this n‐type doping strategy breaks the limitation of poor charge separation caused by the elevated Coulomb interaction.

## 4 Experimental Section

### Chemicals and Materials

Urea (AR, 99%), KCl (GR, 99.8%), MgCl_2_ (99%), CaCl_2_ (99.9%), SrCl_2_ (99.5%), BaCl_2_ (99.5%), H_2_PtCl_6_ **^.^** 6H_2_O (AR, Pt≥37.5%), and triethanolamine (TEOA, AR, 98.0%) were purchased from Aladdin Reagent Company.

### Preparation of KCN and Alkaline Earth Metal Ions Doped KCN

The 10 g of urea powder, 9.5 g of KCl, and 0.5 g of MCl_2_ (M = Mg, Ca, Sr, Ba) were added to a 20 mL of the crucible with a cover (sealed with tin foil) and then heated to 600 ℃ at a rate of 2 ℃ min^−1^ for 3 h in the air. Note: Putting urea on top helped dissolve the chlorides below when the urea melts. After being cooled to room temperature, the products were obtained, washed three times with water, and dried at 60 ℃ under vacuum. According to different alkaline earth metal chlorides MCl_2_ (M = Mg, Ca, Sr, Ba), the final samples were named MKCN, CKCN, SKCN, and BKCN, respectively. KCN doped with different contents of Ca^2+^ ions were prepared by adjusting the amount of CaCl_2_ (0.1–2 g) (keeping the total mass of KCl and CaCl_2_ at 10 g). KCN was synthesized under the same conditions except that no MCl_2_ was added. CN was obtained under the same conditions by direct condensation of urea without KCl and MCl_2_.

## Conflict of Interest

The authors declare no conflict of interest.

## Supporting information

Supporting information

## Data Availability

Research data are not shared.
