# Supplementary material for: Breaking the Limitation of Elevated Coulomb Interaction in Crystalline Carbon Nitride for Visible and Near‐Infrared Light Photoactivity
Source: Adv Sci (Weinh). 2022 Jun 2;9(21):2201677. doi: 10.1002/advs.202201677 (PMC9313543; doi:10.1002/advs.202201677)
Supplement: Supplementary file 1 — Supporting information [file ADVS-9-2201677-s001.pdf]

ADVANCED SCIENCE
Open Access

Supporting Information

for *Adv. Sci.*, DOI 10.1002/advs.202201677

Breaking the Limitation of Elevated Coulomb Interaction in Crystalline Carbon Nitride for Visible and Near-Infrared Light Photoactivity

*Guoqiang Zhang, Yangsen Xu, Muhammad Rauf, Jinyu Zhu, Yongliang Li, Chuanxin He, Xiangzhong Ren, Peixin Zhang and Hongwei Mi**

Supporting Information

Breaking the limitation of elevated Coulomb interaction in crystalline carbon nitride for visible and near-infrared light photoactivity

*Guoqiang Zhang, Yangsen Xu, Muhammad Rauf, Jinyu Zhu, Yongliang Li, Chuanxin He, Xiangzhong Ren, Peixin Zhang, and Hongwei Mi**

Dr. G. Zhang, Dr. M. Rauf, J. Zhu, Prof. Y. Li, Prof. C. He, Prof. X. Ren, Prof. P. Zhang, Prof. H. Mi
College of Chemistry and Environmental Engineering, Shenzhen University, Shenzhen, Guangdong, 518060, PR China
Email: milia807@szu.edu.cn

Dr. Y. Xu
Institute of Information Technology, Shenzhen Institute of Information Technology, Shenzhen, Guangdong, 518172, PR China

1.1. Characterizations

X-ray photoelectron spectrum (XPS) analyses were performed on an ESCALAB 250Xi spectrometer with an Al-K_α (1486.6 eV) achromatic X-ray source. Brunauer-Emmett-Teller (BET) specific surface area was measured using a Micrometrics ASAP 2020 HD88 Surface Area and Pore Size Analyzer. The UV-Vis absorption spectra were recorded on a UV-3600 scanning spectrophotometer (Shimadzu). Inductively coupled plasma spectrometry (ICP-OES) was performed on Ultima 2, Horiba. The solid-state ^{13}C NMR spectra were recorded on a Bruker Avance II instrument in cross-polarization magic-angle spinning sequence mode. The Fourier transform infrared (FTIR) spectra were recorded on a Nicolet iz10 spectrometer. Steady-state and temperature-dependent fluorescence emission spectra were recorded on a LabRAM HR Evolution spectrograph. Transient state fluorescence spectra were recorded on an Edinburgh instruments FS5 fluorescence spectrometer. The crystalline structure was recorded by using an X-ray diffractometer (XRD) (Empyrean), using Cu K_α radiation (λ = 1.54056 A). Transmission electron microscope (TEM) images were taken using a JEOL JEM-2010 operated at 200 kV. Scanning electron microscope (SEM) images are measured on Hitachi SU8010.

1.2. Photocatalytic H_2 production Measurements

The 50 mg of samples added with H_2PtCl_6 (3 wt% Pt) is placed into a 50 mL of TEOA solution (10 vol%) in a closed gas circulation system (Beijing

Perfectlight, Labsolar-6A). The visible-light irradiations were obtained from a 300 W Xe lamp (Beijing Perfectlight, PLS-SXE300) with UVCUT-420, UVCUT-500 and UVCUT-700 filters. All tests are controlled at 5 °C by recirculating cooling water system. The evolved gases are detected *in situ* by using an online gas chromatograph (GC9790II, Fuli) equipped with a thermal conductivity detector (TCD). In order to investigate the relationship between the mass of photocatalysts and H_2 production rates, we also conducted tests under different amount of photocatalysts (10-200 mg) while keeping the Pt load at 3wt%.

1.3. AQE calculations of H_2 production

The 50 mg of samples added with H_2PtCl_6 (3 wt% Pt) is placed into a 100 mL of TEOA solution (10 vol%) with 3 wt% KCl in a closed gas circulation system. The catalyst solution is irradiated by a 300 W Xe lamp applying bandpass filters (center at 420, 450, 475, 500, 550, 600, 650 and 700 nm) for 1 h. The xenon lamp equipped with AM 1.5G filter is used to simulate sunlight to measure the solar to hydrogen conversion efficiency (STH). All tests are controlled at 25 °C by recirculating cooling water system. The average optical power densities are measured by an FZ-A spectroradiometer (Photoelectric Instrument Factory of Beijing Normal University). The apparent quantum efficiency (AQE) was calculated from equation:

$$\mathrm{AQE} = \frac{2\ \times the\ number\ of\ evolved\ H_2\ molecules}{the\ number\ of\ incident\ photons} \times 100\%$$

1.4. fs-TA spectroscopy measurements

The ultrafast fs-TA measurements were performed on a Helios (Ultrafast systems) spectrometers using a regeneratively amplified femtosecond Ti: sapphire laser system (Spitfire Pro-F1KXP, Spectra-Physics; frequency, 1 kHz; max pulse energy, ~8 mJ; pulse width, 120 fs) at room temperature. Finally, the data was analysed through commercial software (Surface Xplorer, Ultrafast Systems).

1.5. Hall measurement

The samples were pressed by the tablet press, and then the resistance of the pressed samples was tested with a multimeter. The test current was set to -200 to 200 mA depending on the resistance of the pressed sample. The samples were loaded into a four-probe table and tested with van der Pauw Hall measurement system (Accent HL5500) under the magnetic field strength of 0.5 T.

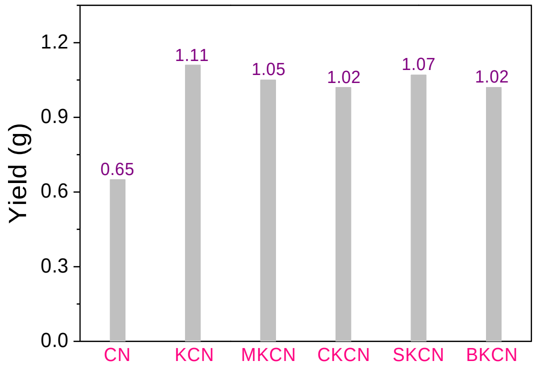

Figure S1. The yield of CN, KCN, MKCN, CKCN, SKCN and BKCN samples.

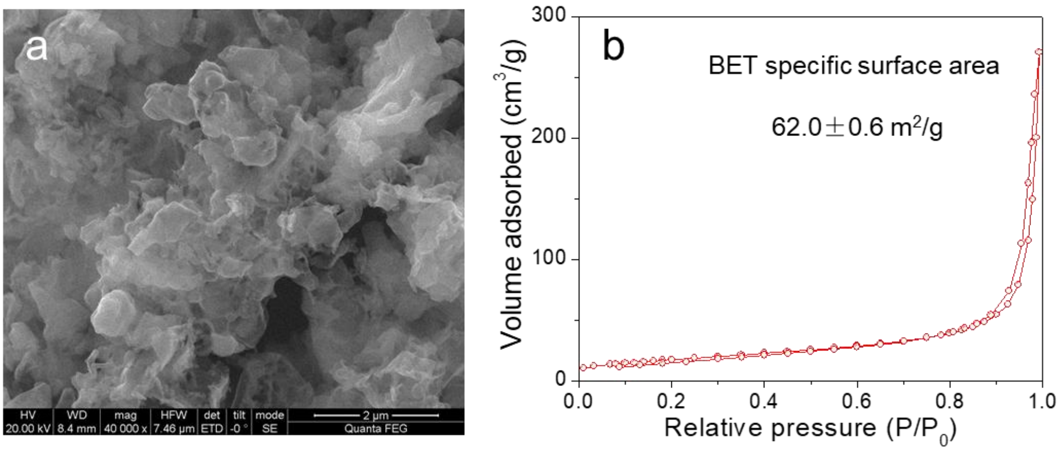

Figure S2. SEM image (a) and the N_2 adsorption-desorption isotherms (b) of CN.

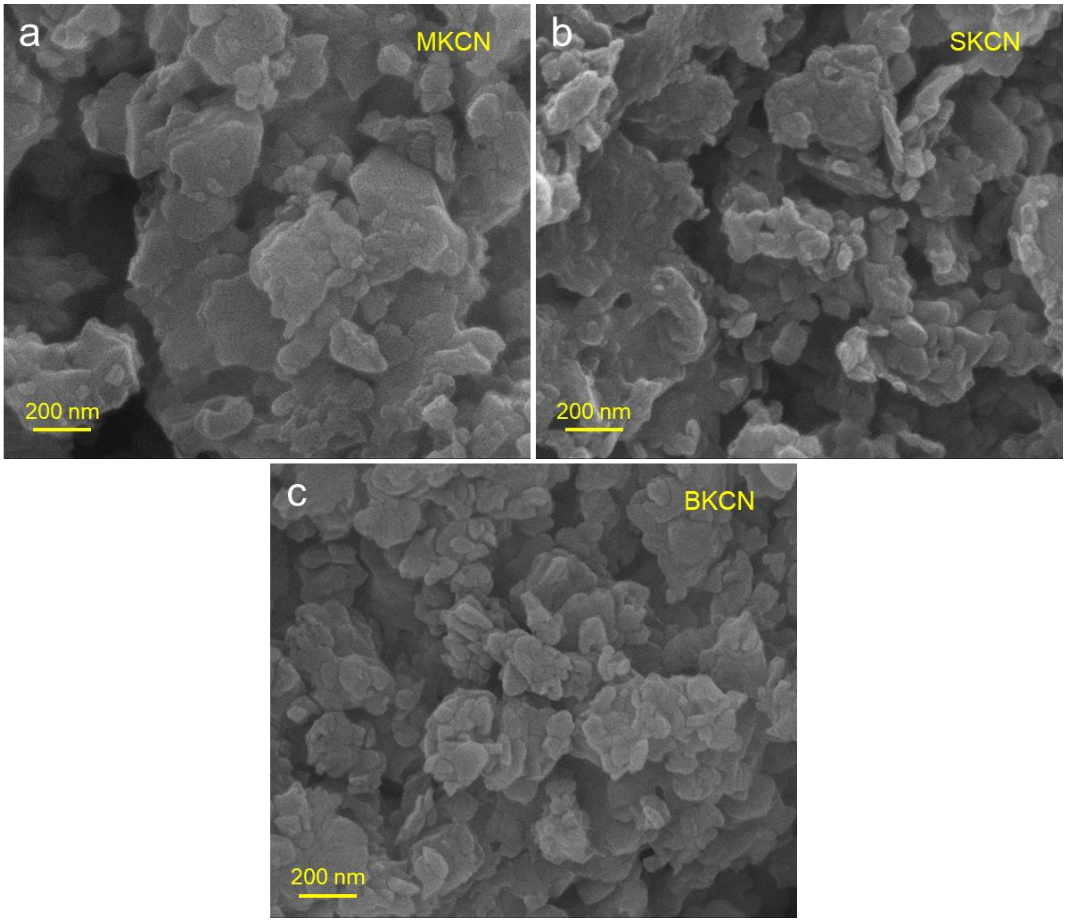

Figure S3. SEM images of MKCN, SKCN and BKCN.

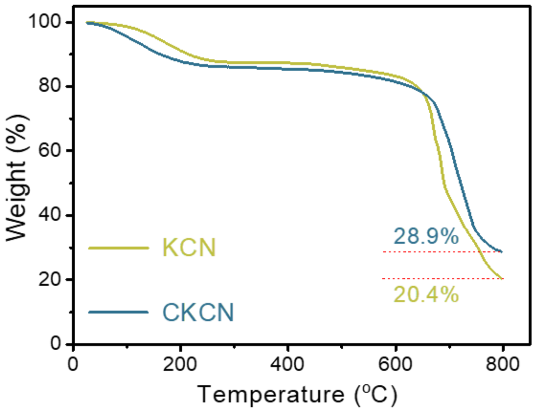

Figure S4. The thermogravimetry of KCN and CKCN.

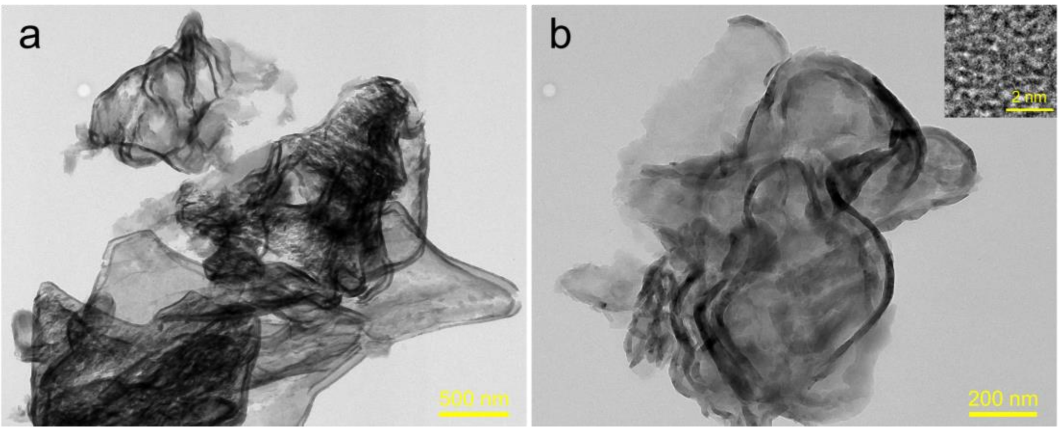

Figure S5. TEM images (a-b) of CN. Inset in (b) is the HR-TEM image.

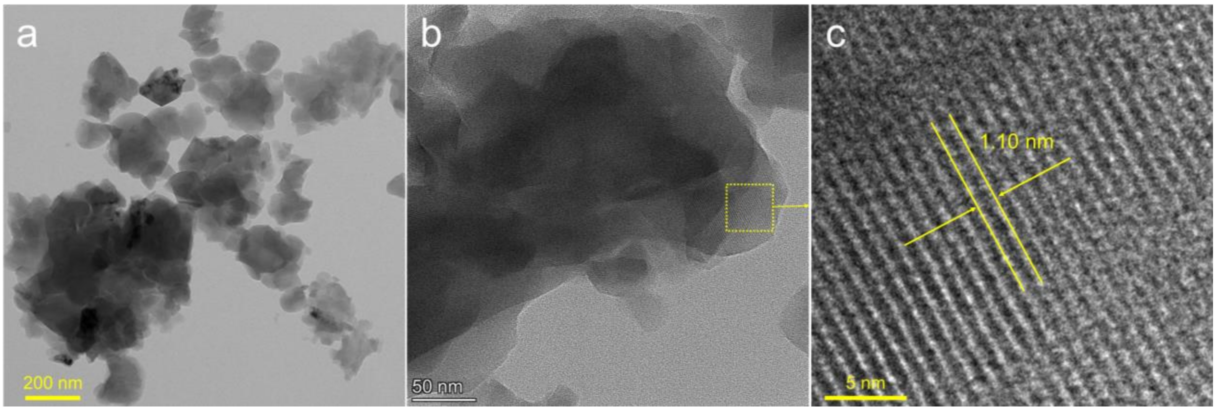

Figure S6. TEM images (a-b) and HR-TEM image (c) of KCN.

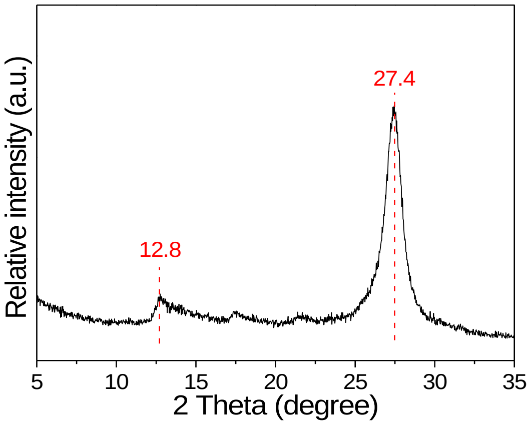

Figure S7. XRD pattern of CN.

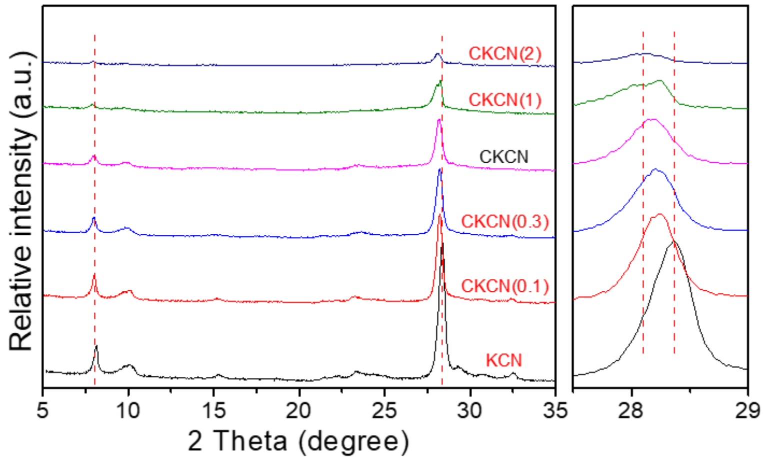

Figure S8. The XRD patterns of KCN doped with different contents of Ca^{2+} ions.

Table S1. Elemental analysis of KCN and CKCN.

Elemental analysis	KCN	CKCN
C (wt%)	24.91	24.99
N (wt%)	41.78	41.71
H (wt%)	1.84	2.45
C/N (molar ratio)	0.696	0.699

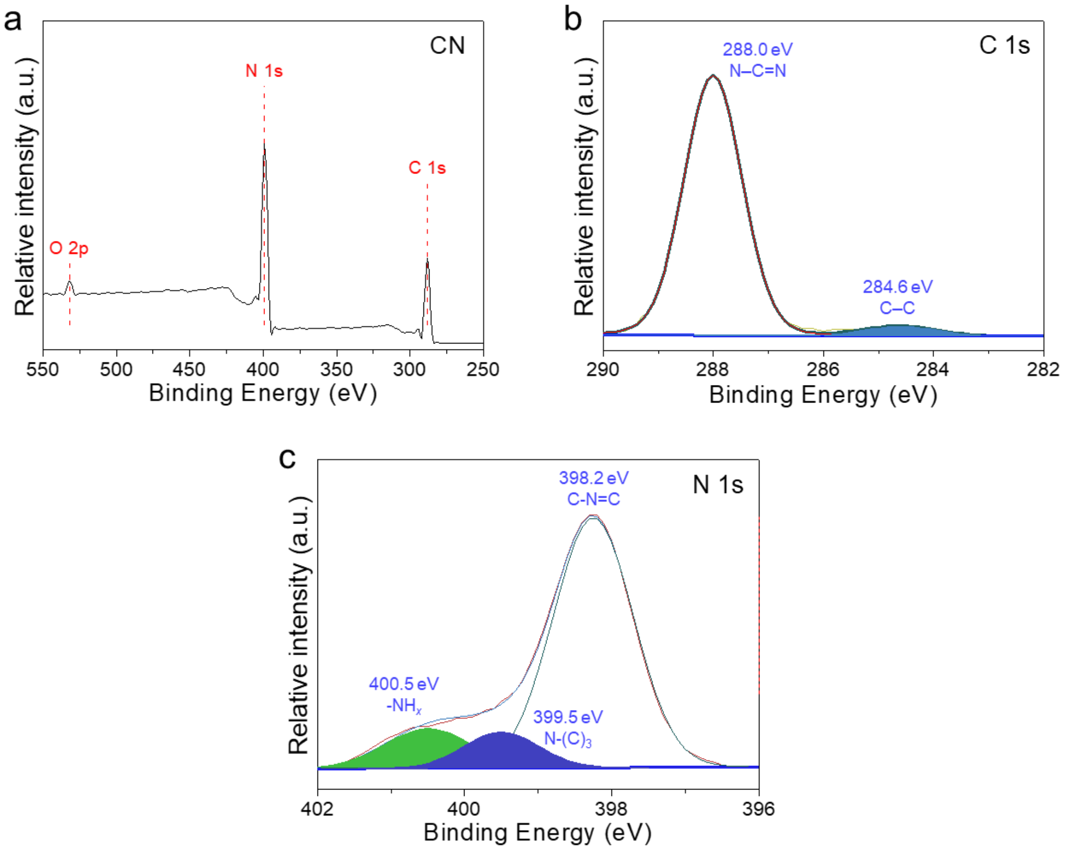

Figure S9. The survey XPS spectra (a) and high-resolution XPS spectra (b-c) of CN.

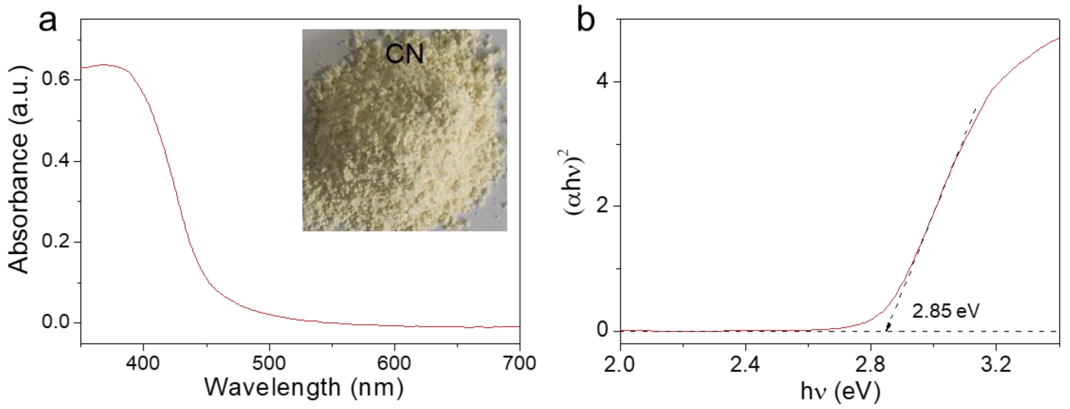

Figure S10. The UV-Vis DRS spectra (a) and Tauc plot (b) of CN. Inset in (a) is the optical photograph.

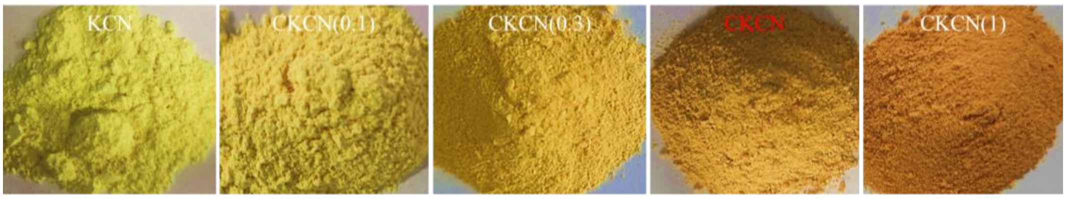

Figure S11. Optical photos of KCN doped with different contents of Ca^{2+} ions.

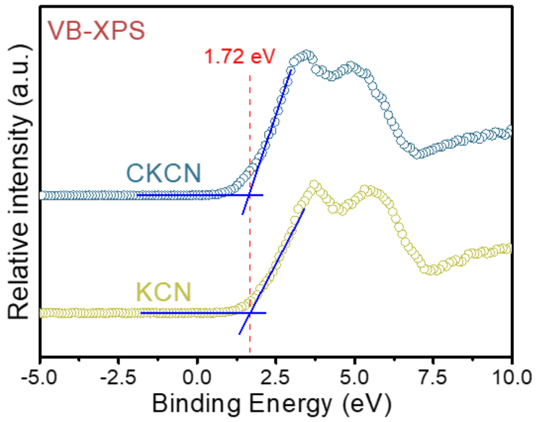

Figure S12. The valence band XPS spectra of KCN and CKCN.

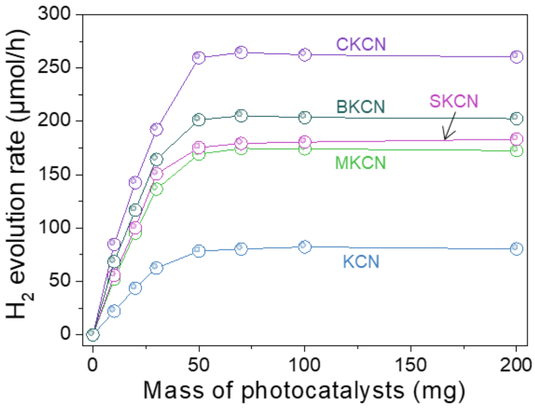

Figure S13. H_2 production rates of photocatalysts with different mass under λ > 420 nm irradiations.

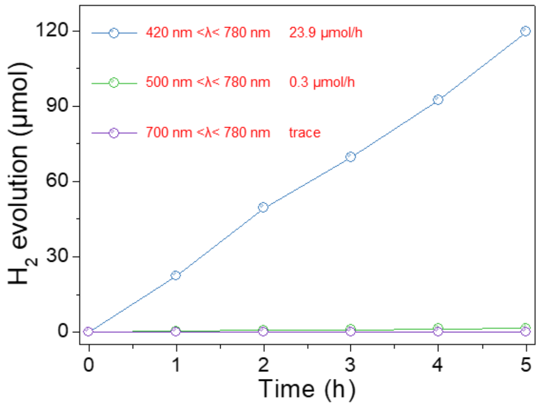

Figure S14. H_2 production of CN under 420 nm <λ< 780 nm, 500 nm <λ< 780 nm and 700 nm <λ< 780 nm irradiations.

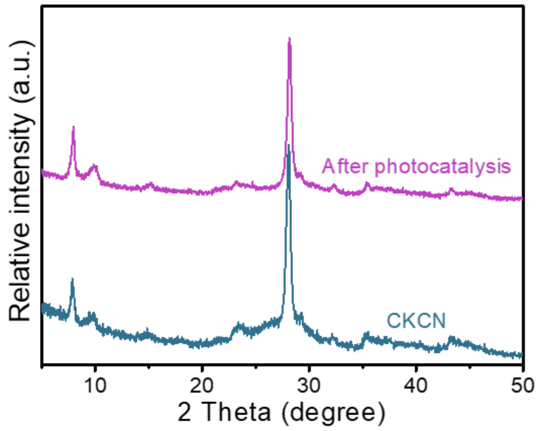

Figure S15. XRD patterns of CKCN before and after photocatalysis.
